# Hepatocyte growth factor-modified hair follicle stem cells ameliorate cerebral ischemia/reperfusion injury in rats

**DOI:** 10.1186/s13287-023-03251-5

**Published:** 2023-02-13

**Authors:** Hao Tang, Xuemei Zhang, Xiaojun Hao, Haitong Dou, Chendan Zou, Yinglian Zhou, Bing Li, Hui Yue, Duo Wang, Yifei Wang, Chunxiao Yang, Jin Fu

**Affiliations:** 1 Department of Neurology, The Second Affiliated Hospital of Harbin Medical University, No.246 Xuefu Road, Nangang District, Harbin, 150086 Heilongjiang China; 2 Department of Biochemistry and Molecular Biology, Harbin Medical University, No.157 Baojian Road, Nangang District, Harbin, 150086 Heilongjiang China

**Keywords:** Hair follicle stem cells, Ischemic stroke, Microglia, Blood–brain barrier, Hepatocyte growth factor

## Abstract

**Background:**

Hair follicle stem cells (HFSCs) are considered as a promising cell type in the stem cell transplantation treatment of neurological diseases because of their rich sources, easy access, and the same ectoderm source as the nervous system. Hepatocyte growth factor (HGF) is a pleiotropic cytokine that shows neuroprotective function in ischemic stroke. Here we assessed the therapeutic effects of HFSCs on ischemic stroke injury and the synthetic effect of HGF along with HFSCs.

**Methods:**

Rat HFSCs were intravenously transplanted into a middle cerebral artery ischemia/reperfusion (I/R) rat model. Neurological scoring and TTC staining were performed to assess the benefits of HFSC transplantation. Inflammatory cytokines, blood–brain barrier integrity and angiogenesis within penumbra were estimated by Western blot and immunohistochemistry. The differentiation of HFSCs was detected by immunofluorescence method 2 weeks after transplantation.

**Results:**

HFSC transplantation could significantly inhibit the activation of microglia, improve the integrity of blood–brain barrier and reduce brain edema. Moreover, the number of surviving neurons and microvessels density in the penumbra were upregulated by HFSC transplantation, leading to better neurological score. The combination of HFSCs and HGF could significantly improve the therapeutic benefit.

**Conclusion:**

Our results indicate for the first time that HGF modified HFSCs can reduce I/R injury and promote the neurological recovery by inhibiting inflammatory response, protecting blood–brain barrier and promoting angiogenesis.

**Supplementary Information:**

The online version contains supplementary material available at 10.1186/s13287-023-03251-5.

## Introduction

Ischemic stroke is one of the leading causes of death and long-term disabilities [1, 2]. In 2019, about 6.6 million people died of stroke, of which ischemic stroke accounted for 87% of all cases [3, 4]. Ischemic stroke could be caused by various reasons of cerebral artery obstruction such as arteriosclerotic plaque, small vessel lesions and cardiogenic embolism [5, 6]. After the cerebral artery obstruction, pathological changes such as ischemia, hypoxia, edema and neuron apoptosis may occur in brain, leading to tissue necrosis and neurological dysfunction. Thrombolysis therapy within 4.5 h after the onset of stroke, including thrombolytic drugs and mechanical thrombectomy devices, is the most effective method to regain blood flow and rescue brain tissue for ischemic stroke [7, 8]. Although some patients have a favorable prognosis, many patients may still have varying degrees of neurological disorders due to complications such as ischemia/reperfusion (I/R) injury. Moreover, numerous patients who cannot receive thrombolysis therapy due to the contraindication of systemic thrombolysis or time limitation like exceeding 4.5 h after the onset of stroke will suffer more serious brain tissue damage and dysfunction [9].

In order to improve the prognosis of patients with ischemic stroke, many neuroprotective therapeutic studies have been carried out. Although more than 50 neuroprotective drugs have entered clinical trials, including calcium antagonists, glutamate antagonists, glutamate release inhibitors, GABA receptor agonists, free radical scavengers, cell membrane stabilizers and so on [10, 11], unfortunately, most drugs do not work well in clinical trials [12, 13]. Stem cell transplantation is one of the most innovative and attractive experimental therapies. It realizes cell replacement and regulates the damaged or inflammatory environment through a variety of mechanisms [14–16]. Hair follicle stem cells (HFSCs) are considered as a promising cell type for transplantation treatment in neurological diseases. HFSCs were found in mouse hair follicles which were characterized by expressing neural progenitor cell marker nestin instead of keratin cytokeratin [17]. HFSCs can differentiate into neurons, glial cells, smooth muscle cells, keratinocytes and melanocytes in vitro. McKenzie et al*.* had found HFSC transplantation was involved in the formation of the sciatic nerve myelin in mice with hereditary myelin basic protein deficiency [18]. Moreover, after engrafted into brain, HFSCs migrated to the forehead and generated different neural phenotypes: immature neurons and well differentiated astrocytes [19–21]. However, the role and mechanism of HFSCs in ischemic stroke is still lack of evidence.

Hepatocyte growth factor (HGF) is a powerful pleiotropic cytokine that performs different functions in a variety of biological processes including cell motility, angiogenesis and anti-apoptosis [22–24]. The neuro-regenerative effects of HGF have been explored in animal models, such as spinal cord injury and acute stroke in vivo [24, 25]. Recent studies have shown that HGF could protect blood–brain barrier (BBB) integrity, attenuate brain edema and promote endogenous repair and functional recovery in rodent models after cerebral ischemia [26]. Clinical trials of HGF have also been carried out in the treatment of coronary and peripheral arterial ischemia, with good tolerance and effect [27–29]. However, HGF is difficult to become an independent therapeutic method due to the problems of short serum half-life, difficulty in entering the central nervous system (CNS) through the systemic route [30].

In the present study, we aimed to assess whether HFSCs and HGF-modified HFSCs play a therapeutic role in ischemic stroke. The neurological function was evaluated after HFSC transplantation in cerebral I/R injury rat models. We also assessed the mechanisms underlying the neuro-protective effects of HFSCs including inflammation alleviation, BBB protection, angiogenesis promotion and neuron apoptosis inhibition.

## Materials and methods

### HFSCs preparation

The hair follicles were harvested by enzyme digestion followed by mechanical dissection [17]. To isolate the vibrissa follicles, the upper lip containing the vibrissa pad of male SD rats (21–26 g) was cut and digested with 0.1% collagenase in DMEM (both from Gibco BRL, Gaithersburg, MD, USA). Then the vibrissa follicles were gently plucked out of the pad and seeded in 24-well tissue-culture dishes (Corning, NY, NYS, USA). The hair follicles were cultured in DMEM containing 10% fetal bovine serum (ScienCell, Santiago, CA, USA) and 1% penicillin & streptomycin (Gibco BRL, Gaithersburg, MD, USA) at 37 °C in a humidified atmosphere with 5% CO_2_. All surgical procedures were performed under a sterile environment. The third passage of cultured HFSCs were transduced (co-culture) with either HGF (Rat HGF Gene ID: 24,446, MOI = 80) or blank lentivirus at a multiplicity of infection at least 50% for 72 h. The transduction efficiency was evaluated under a fluorescence microscope (Leica DMI 4000 B, Wetzlar, Germany) by the equation of (EGFP positive cells/total cells) * 100%. The transduced cells were sorted by puromycin dihydrochloride (1 µg/mL; Thermo Fisher Scientific, Waltham, MA, USA) for three passages. The HFSCs identification was completed by fluorescence-activated cell sorting (FACS) (BD FACS Canto II, Franklin Lakes, NJ, USA) with the antibodies of CD29 (0.5 mg/mL; 561,796, BD Pharmingen, Franklin Lakes, NJ, USA), CD90 (0.2 mg/mL; 551,401, BD Pharmingen, NJ, USA), CD31 (1:200 dilution; Miltenyi Biotec, Bergisch Gladbach, Germany) and CD45 (0.2 mg/mL; 559,135, BD Pharmingen, Franklin Lakes, NJ, USA) [31]. The HGF gene expression and blank lentivirus vectors were commercially provided by Shanghai JiKai Chemical Technology. The viral vector used in the study is shown in Additional file 1: Fig. S1D. For unmodified HFSCs tracing, the non-cytotoxic membrane dye PKH-67 (Sigma-Aldrich, St Louis, MO, USA) was used before transplantation.

### HFSCs pluripotency evaluation

HFSCs in the third passage were seeded in culture medium at 3 × 10^4^ cells/cm^2^ in six-well tissue culture plates. After incubation for 24 h, the growth medium was changed to osteogenic or adipogenic differentiation medium, according to the manufacturer’s protocol [32]. Cells were then cultured at 37 °C in a humidified atmosphere with 5% CO_2_, and the osteogenic and adipogenic differentiation medium was replaced every 3 days. After 14 days culture, osteogenic and adipogenic differentiation was verified by Alizarin Red (Sigma-Aldrich, St Louis, MO, USA) staining and Oil Red O (Sigma-Aldrich, St Louis, MO, USA) staining (Additional file 1: Fig. S1H, I).

### Focal cerebral ischemia/reperfusion animal model

Male Sprague–Dawley (SD) rats weighing 280 ± 10 g were purchased from the animal center of the Second Affiliated Hospital of Harbin Medical University. All the rats were maintained in a barrier facility, and all experimental studies were carried out in accordance with the relevant ethical guidelines approved by the Experimental Center of the Second Affiliated Hospital of Harbin Medical University. Rats were anesthetized by 0.3% pentobarbital sodium (50 mg/kg body weight) intraperitoneally and subjected to right middle cerebral artery occlusion (MCAO) for 1 h following reperfusion [33, 34]. Briefly, after anesthetized, neck vessels were exposed and the right middle cerebral artery (MCA) was occluded with a 0.38 mm rounding tip suture by inserting it through the right external carotid artery (ECA) and gently advancing into the internal carotid artery (ICA). After 60 min occlusion, reperfusion was achieved by slowly pulling the suture back. The incision on the neck was sutured and the animals were allowed to recovery. Body temperature was maintained at (37 ± 0.5) °C by using a thermostat-controlled heating pad throughout the procedure. TTC staining and Nissl staining was performed to confirm the establishment of the model (Additional file 2: Fig. S2). The establishment results of HGF over-expressed HFSCs and focal cerebral ischemia–reperfusion rat model were in the supplementary results.

### Intravenous transplantation of HFSCs

A total of 120 rats were included and randomly divided into 5 groups: #1 Control group (healthy rats), #2 Saline group (I/R + Saline), #3 HFSCs group (I/R + HFSCs), #4 HFSCs/Vehicle group (I/R + HFSCs/Vehicle) and #5 HFSCs/HGF group (I/R + HFSCs/HGF). Rats in the #1 Control group were healthy and non-operated, while the rats in the other groups were subjected to right middle cerebral occlusion and reperfusion. HFSCs groups (#3 #4 #5) were subjected to modified or unmodified HFSCs transplantation (1*10^6^ cells dispersed in 1 mL saline) via caudal vein injection 24 h after reperfusion. The animals in #2 Saline group were given equivalent volume of saline in the same manner. At 7 days after cell transplantation, 90 animals were anesthetized by 0.3% pentobarbital sodium (150 mg/kg body weight) intraperitoneally for euthanasia and dedicated to 2,3,5-Triphenyltetrazolium chloride (TTC) staining, histological staining and western blot assay (*n* = 6). The other animals were allowed to survive 2 weeks after transplantation for histological staining (*n* = 6).

### Neurological scores

After cerebral I/R surgery, neurological scores were evaluated daily as described in Additional file 4: Table S1 [35]. Each animal’s scores were estimated three times for consistency. A score of 15 corresponds to a normal neurological status.

### TTC staining

TTC staining was used to measure the infarct volume as described previously [36]. Briefly, brains were cut into 5 coronal brain slices (2 mm thick) with a brain matrix (WPI-Europe, Aston, Stevenage, UK), and stained in 1% TTC (Sigma-Aldrich, St Louis, MO, USA) in 0.1 mol/L phosphate buffer for 30 min at 37 °C. The normal tissue was stained red, whereas the infarcted tissue remained unstained (white). The infarct zone was analyzed by Image-Pro Plus 6.0 software (Media Cybernetic, Bethesda, MD, USA). The infarct volume was calculated by the equation [(contralateral hemisphere volume—non-infarcted volume of ipsilateral hemisphere)/contralateral hemisphere volume] * 100% to avoid the influence of brain edema [33].

### Nissl staining

Nissl staining was used to assay the morphological variation of neurons [37]. The animals under anesthesia were perfused transcardially with saline and followed by 4% paraformaldehyde in 0.01 M PBS (pH 7.4). Brains were removed and post-fixed with the same fixative for 48 h, and cryoprotected in 30% sucrose in PBS for at least 48 h at 4 °C, then embedded in OCT (Sakura Finetek, Torrance, CA, USA) and stored at -80 °C. The coronal brain slices (10 µm thick) were cut between the optic chiasma and the cerebral caudal end in a cryostat (Thermo Scientific Microm HM560, Waltham, MA, USA). After air dried, the sections were immersed in cresyl violet acetate (Sigma Aldrich, St Louis, MO, USA) for 2 h at 37 °C, then conducted dehydration and hyalinization.

### Brain water content

Brain water content was measured as reported [38]. The brains were quickly separated into the left and right cerebral hemispheres and weighed (wet weight). Brain samples were then dried in an oven at 120 °C for 48 h and weighed again (dry weight). The percentage of water content was calculated as [(wet weight—dry weight)/wet weight] * 100%.

### TUNEL-positive cells assessment

A cell in situ death detection kit (Fluorescein dUTP Kit; Roche Inc., Indianapolis, IN, USA) was used to evaluate the apoptotic neural cells in the ischemic hemisphere, according to the instruction of manufacturer [39]. Five fields were selected from the surrounding infarction area by a laser confocal microscope (Zeiss LSM800; Carl Zeiss, Jena, Germany). The number of TUNEL-positive nuclei (green) and the total number of nuclei (blue) were scored respectively, and the ratio of the two values was calculated using Image-Pro Plus 6.0 software. The mean ratio of five fields was used for statistic.

### Immunohistochemical staining

The sections were immersed in 0.3% H_2_O_2_ for 30 min to block endogenous peroxidase. Following, the sections were blocked with normal goat serum (abs933, Absin, Shanghai, China) for 30 min at 25 °C, then incubated with primary rabbit anti-doublecortin (DCX, 1:100 dilution; ab18723, Abcam, Cambridge, UK), mouse anti-neuron-specific nuclear protein (NeuN, 1:100 dilution; MAB377, Millipore Corp, Billerica, MA, USA) or Ionized calcium-binding adaptor molecule 1 (Iba-1, 1:100 dilution; ab153696, Abcam, Cambridge, UK) antibodies overnight at 4 °C. The sections were incubated with horseradish peroxidase-linked anti-rabbit or anti-mouse IgG (1:500 dilution; Cell Signaling Technology, Inc., Beverly, MA, USA) for 60 min at 25 °C, and visualized with DAB substrate kit (Cell Signaling Technology, Inc., Beverly, MA, USA). After washed by PBS, sections were counterstained with hematoxylin, then conducted dehydration and hyalinization. The number of positive cells was observed and assessed under an optical microscope. The ratio was calculated using Image-Pro Plus 6.0 software.

### Immunofluorescent staining

After blocking 5% normal goat serum at 25 °C for 30 min, the slices were incubated with primary antibodies diluted in PBST overnight at 4 °C. The primary antibodies were as follows: rabbit anti-DCX and mouse anti-NeuN to label neurons, rabbit anti-vWF (1:100 dilution; ab6994, Abcam, Cambridge, UK) to label endothelial cells. After rinsing with PBS, brain sections were incubated with rhodamine conjugated second antibodies for 1.5 h at 25 °C. The nuclei were stained with 4’-6-diamidino-2-phenylindole (DAPI, Sigma-Aldrich, St Louis, MO, USA). Co-localization of EGFP or PKH67, with neural specific markers and DAPI was observed by laser scanning confocal microscopy at wavelengths of 594 nm (red), 488 nm (green) and 405 nm (blue).

### Western blot assay

Frozen samples were homogenised with tissue extraction buffer containing protease inhibitors, then centrifuged at 12,000 rpm at 4 °C for 10 min. The supernatants were collected, and the protein concentration was measured via BCA protein assay kit. Protein samples (40 µg) were separated by SDS–polyacrylamide gels electrophoresis (Tanon EPS 600, Shanghai, China) at 100 V for 90–120 min and transferred to PVDF membrane with 200 mA at 4 °C for 120 min. Nonspecific binding sites were blocked by preincubating PVDF membrane with 3% nonfat milk in TBS-T for 60 min at 25 °C. Then PVDF membranes were incubated overnight at 4 °C with the primary antibodies against β-actin (1:2000 dilution; sc-47778, Santa Cruz Biotechnology, Santa Cruz, VA, USA), HGF (1:200 dilution; ab83760, Abcam, Cambridge, UK), Bcl-2 (1:1000 dilution; sc-7382, Santa Cruz Biotechnology, Santa Cruz, VA, USA), Bax (1:1000 dilution; sc-7480, Santa Cruz Biotechnology, Santa Cruz, VA, USA), cleaved Caspase-3 (1:500 dilution; 9661, Cell Signaling Technology, Inc., Beverly, MA, USA), occludin (1:50 dilution; sc-133256, Santa Cruz Biotechnology, Santa Cruz, CA, USA), zonula occluden 1 (ZO-1, 1:50 dilution; sc-33725, Santa Cruz Biotechnology, Santa Cruz, CA, USA), TNF-α (1:2000 dilution; AF-510-NA, R&D systems, Minneapolis, MN, USA), and IL-4 (1:2000 dilution; AF-504-NA, R&D systems, Minneapolis, MN, USA). After rinsing with TBS-T for three times, PVDF membranes were further incubated with horseradish peroxidase (HRP)-conjugated secondary antibodies (1:5000 dilution; Cell Signaling Technology, VT, USA) and treated with the enhanced chemiluminescence (ECL) Plus Western Blotting Detection Kit. Finally, immunoblot images were captured using the Omega-Lum G imaging system (ChemiScope 6300, Clinx Science Instruments, Shanghai, China). Bands were calculated by Image-Pro 6.0 software for semiquantification. All the values were normalized by equal loading control.

### Statistical analysis

All parameters were expressed as mean ± Standard deviations (SDs). The image density was measured using the Image-Pro Plus 6.0 software (Media Cybernetic, Bethesda, MD, USA). Statistical analysis was performed using GraphPad Prism 6.0 (GraphPad Software, Inc., San Diego, CA, USA). The data were analyzed using one-way ANOVA followed by Tukey’s test for multiple comparisons. Values with *P* < 0.05 were considered statistically significant.

## Results

### HFSC transplantation decreased infarct volume and improved neurological outcome in rat I/R model

**Fig. 1:**
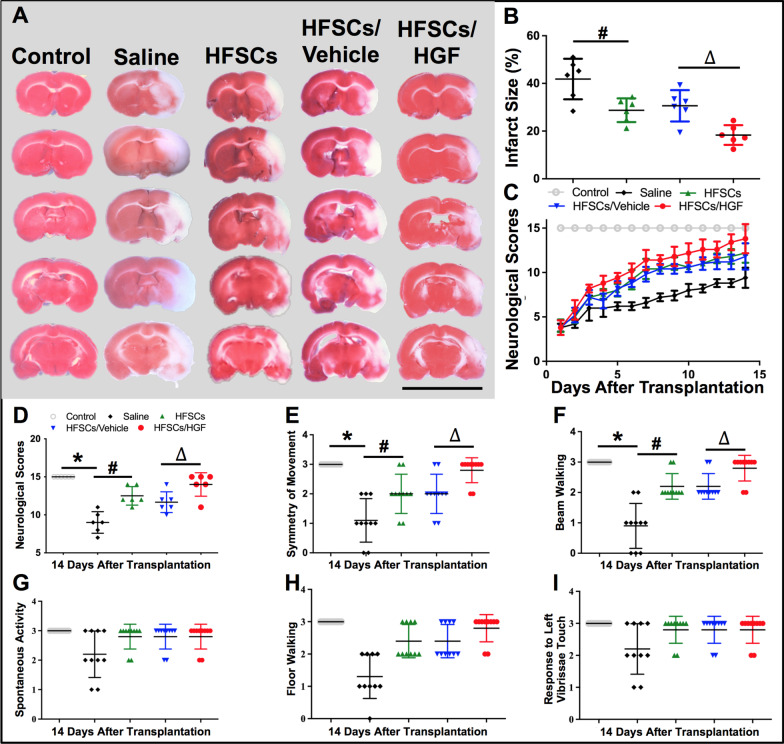
HFSCs and HFSCs/HGF decreased infarct volume and promoted neurological recovery. **A** Representative images of TTC staining in all groups, wherein normal tissue was stained red, while infarction area remained white. Scale bar = 20 mm. **B** Quantitative analysis of infarct size. **C** Dynamic changing of neurological scores with cell transplantation days in different groups. Score of 15 corresponds to a normal neurological status. **D** Total neurological scores in different groups at 2 weeks after cell transplantation. **E** Symmetry of movement scores. **F** Beam walking scores. **G** Spontaneous activity scores. **H** Floor walking scores. **I** Response to left vibrissae touch scores. Values are the mean ± SD. *P < 0.05 Saline group vs. Control group, ^#^*P* < 0.05 HFSCs group vs. Saline group, ^∆^*P* < 0.05 HFSCs/HGF group vs. HFSCs/Vehicle group, *n* = 6

To investigate whether intravenously transplanted HFSCs could decrease infarct volume in cerebral I/R models, TTC staining was conducted at 7 days after cell transplantation. The representative images and statistical analysis of TTC staining in all groups are shown in Fig. 1A and B. Compared with the Saline group, HFSCs and HFSCs/Vehicle significantly reduced I/R-elicited cerebral infarction, while the HFSCs/HGF group exhibited the smallest infarct volume.

Neurological outcomes were evaluated daily after I/R surgery by neurological scoring system. As shown in Fig. 1C, the neurological scores in the Saline group decreased obviously compared with the Control group, while HFSCs and HFSCs/Vehicle treatment promoted neurological dysfunction recovery. Moreover, the results revealed a much faster and better recovery outcome in the HFSCs/HGF group. The total neurological scores of different groups at 2 weeks after treatment are exhibited in Fig. 1D. Among the neurological score subclasses, symmetry of movement (Fig. 1E) and beam walking (Fig. 1F) were two remarkable indexes to reflect the therapeutic effects of cell transplantation. There was no significant difference in spontaneous activity, floor walking and response to vibrissae touch at 2 weeks after I/R surgery (Fig. 1G–I).

Thus, the therapeutic effects of HFSCs and HFSCs/HGF treatment were verified on infarct volume and neurological outcomes. Next, we aimed to further detect the impact of cell treatment on neuron survival.

### HFSC transplantation ameliorated neuron apoptosis in penumbra region

**Fig. 2:**
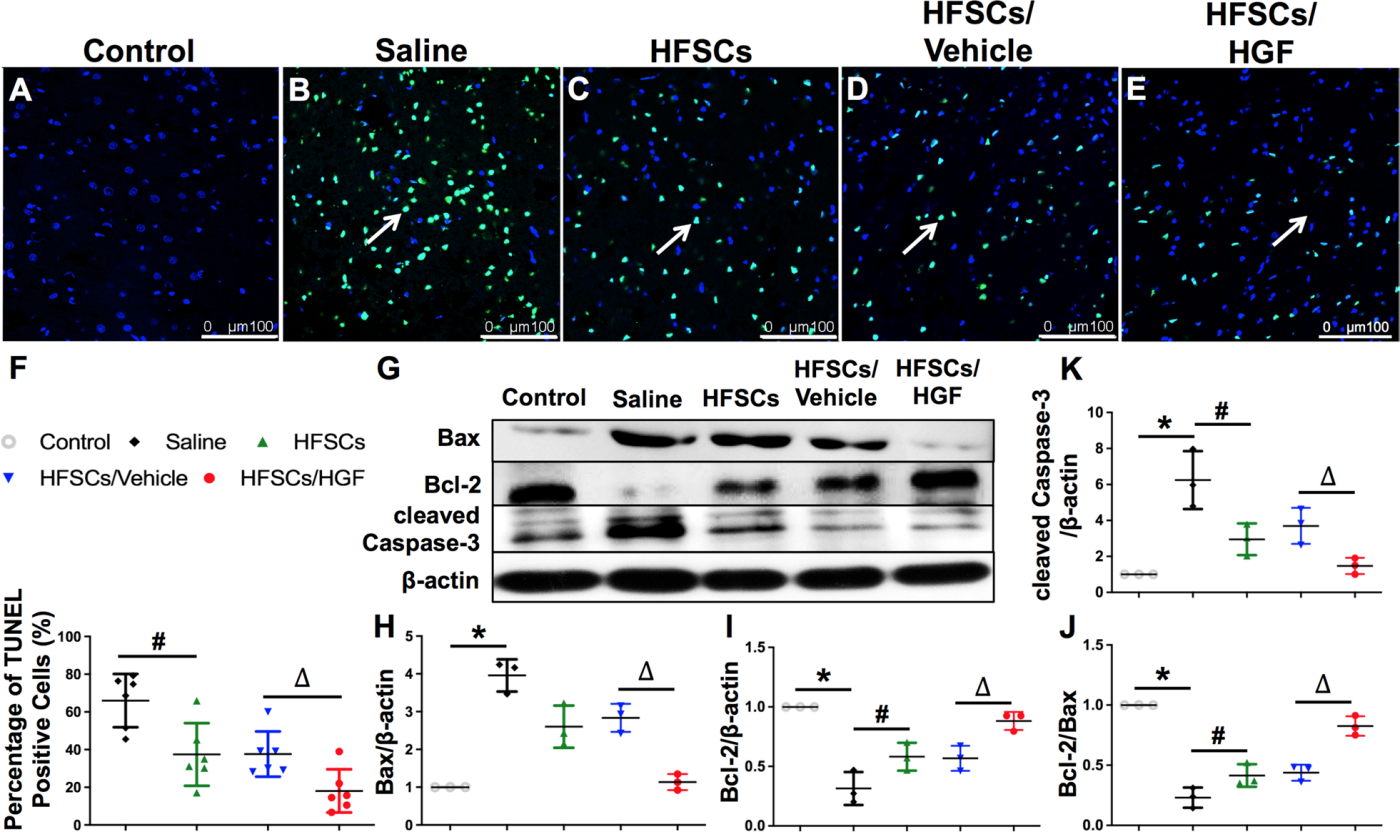
HFSCs and HFSCs/HGF ameliorated neuron apoptosis. **A–E** Representative photographs of TUNEL-positive cells in all groups. Normal nucleus was stained blue and TUNEL-positive cells green (arrows). Tissue samples were collected from the penumbra of ischemic cerebral hemispheres. Scale bar = 100 µm. **F** The quantitative analysis of TUNEL-positive cells in different groups. **G** Representative western blot bands of apoptosis-related proteins in different groups. **H–K** Quantification of Bax, Bcl-2, Bcl-2/Bax and cleaved Caspase-3 expression. All the quantifications were undertaken based on the data of 6 independent experiments and normalized to β-actin. Values are the mean ± SD. *P < 0.05 Saline group vs. Control group, ^#^*P* < 0.05 HFSCs group vs. Saline group, ^∆^*P* < 0.05 HFSCs/HGF group vs. HFSCs/Vehicle group, *n* = 6

To investigate the neuron damage in the ischemic hemisphere, TUNEL in situ kit was used to indicate neuron apoptosis and western blot to detect apoptosis related proteins. Figure 2A–E shows the representative images of TUNEL staining of all groups conducted at 7 days after cell transplantation. The results revealed that the treatment of HFSCs and HFSCs/Vehicle inhibited neuron apoptosis around the ischemic area. Moreover, HFSCs/HGF treatment further decreased the number of TUNEL-positive cells compared to HFSCs groups. The expression of Bax, Bcl-2, and cleaved Caspase-3 were detected by western blot (Fig. 2G). Bcl-2 expression decreased while Bax and cleaved Caspase-3 expression increased in the ischemic hemisphere after cerebral I/R injury. These changes were reversed by HFSCs treatment, while HFSCs/HGF exhibited even stronger influence than HFSCs and HFSCs/Vehicle.

Taken together, the results proved the anti-apoptosis effect of HFSCs in ischemic stroke.

**Fig. 3:**
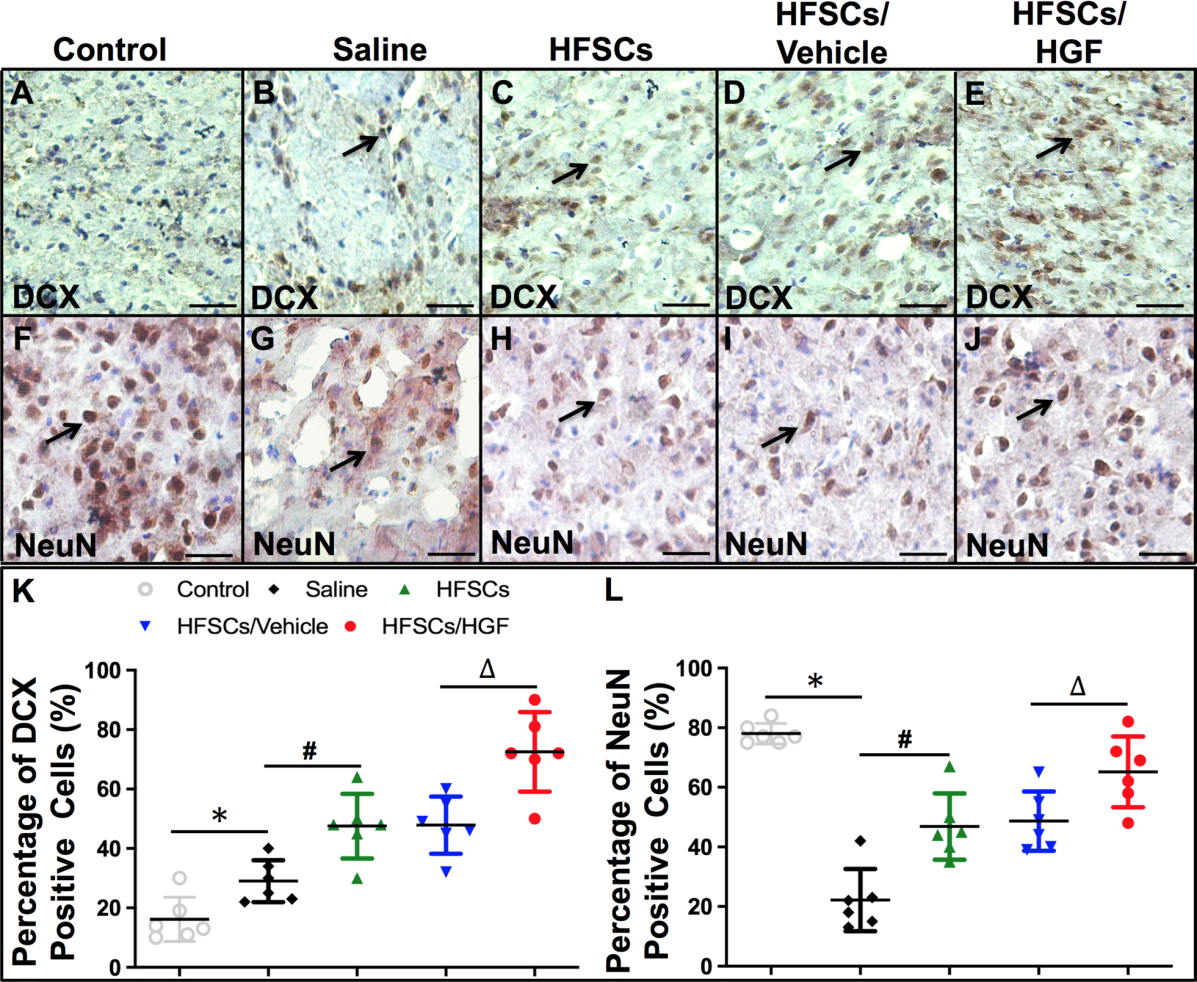
HFSCs and HFSCs/HGF increased the number of neuron cells. **A–E** Representative photographs of DCX-positive cells in the penumbra of all groups (black arrows indicated). Scale bar = 25 µm. **F–J** Representative photographs of NeuN-positive cells in the penumbra of all groups (black arrows indicated). Scale bar = 25 µm. **K** Semi-quantitative data of DCX-positive cells in all groups. **L** Semi-quantitative data of NeuN-positive cells in all groups. Values are the mean ± SD. *P < 0.05 Saline group vs. Control group, ^#^*P* < 0.05 HFSCs group vs. Saline group, ^∆^*P* < 0.05 HFSCs/HGF group vs. HFSCs/Vehicle group, *n* = 6

Immunohistochemical analysis of the neuron specific markers including DCX and NeuN in the penumbra region was performed at 7 days after cell treatment (Fig. 3). DCX, a specific marker of immature neurons [40], is a valuable alternative marker used to measure the levels of neurogenesis [39]. NeuN is a neural marker of mature neurons [41]. Densitometry showed a significant increase in DCX positive cells percentage in HFSCs and HFSCs/Vehicle groups relative to the Saline group, and the HFSCs/HGF group retains the highest percentage among all the treatment groups (Fig. 3K) (*P* < 0.05). Additionally, the percentage of NeuN positive cells was significantly decreased in the Saline group compared to the Control group. HFSCs and HFSCs/Vehicle treatment reversed this decrease remarkably, whereas the highest percentage was presented in HFSCs/HGF group (Fig. 3L) (*P* < 0.05).

These data support the therapeutic effect of HFSCs and HFSCs/HGF treatment for protecting neurons from apoptosis. Nevertheless, we still were curious about how the stem cells affected neuron apoptosis in I/R induced brain injury. As far as we know, after ischemia/reperfusion, the following oxidative cascade and inflammation may break BBB and cause extravascular edema around small vessels, which accelerates tissue ischemia [42]. Meanwhile, cytokines and chemokines released from injured neurons and immunocytes further increase the permeability of BBB, allowing peripheral leukocytes to invade and upregulate inflammatory processes [43]. The crosstalk of I/R-elicited inflammation and brain edema accelerates neuron apoptosis in the penumbra area. Therefore, we estimated the inflammatory reaction, BBB integrity and vascular proliferation to explore the anti-apoptosis effect of HFSCs treatment.

### HFSC transplantation suppressed microglia activation and inflammatory cytokines production

**Fig. 4:**
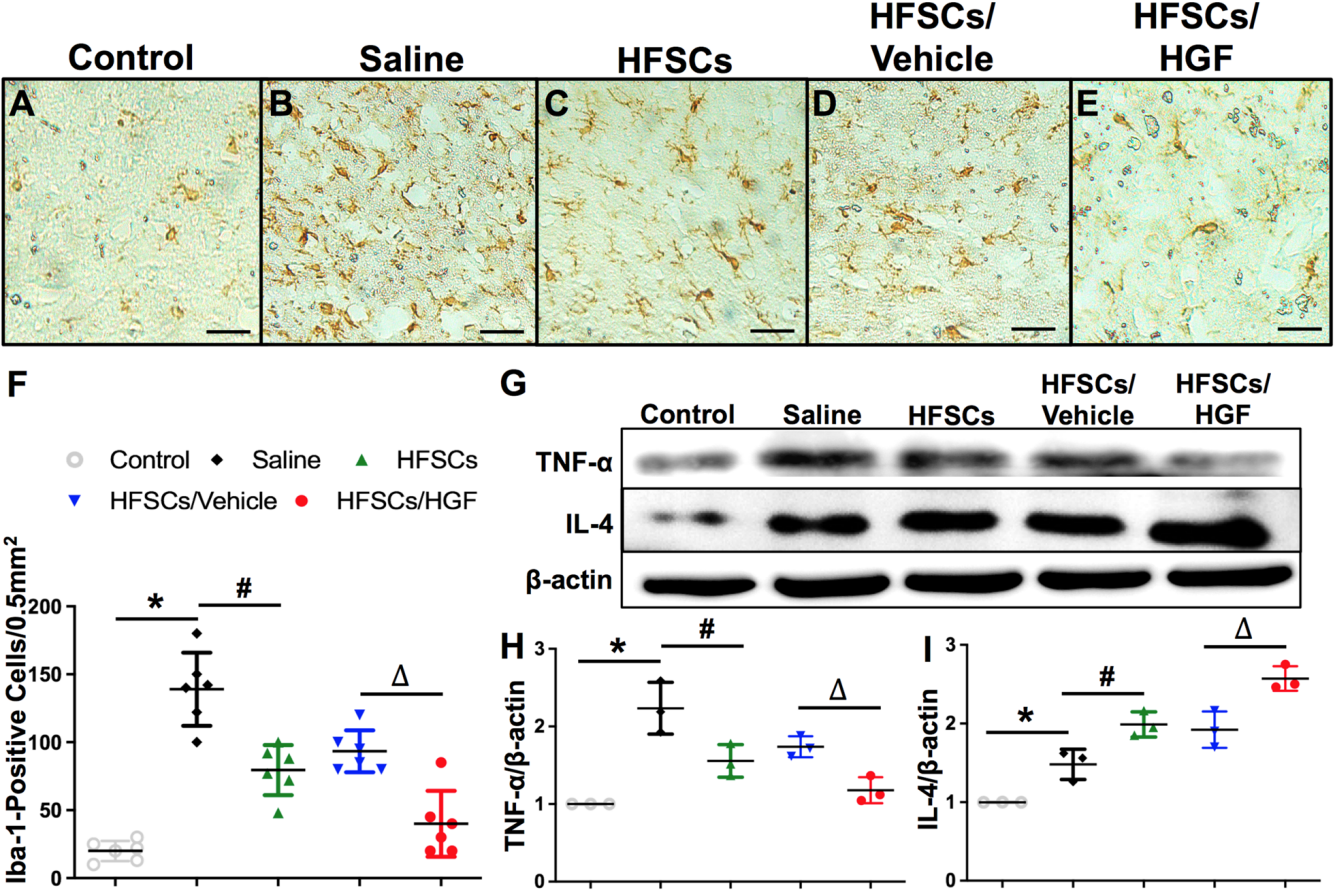
HFSCs and HFSCs/HGF suppressed inflammatory reaction. **A–E** Iba-1 staining in the penumbra at 7 days after cell transplantation. Scale bar = 100 µm. **F** The quantitative analysis of the numbers of Iba-1-positive cells. **G** Representative western blot bands of inflammation-related proteins in different groups. **H** and **I** Quantification of TNF-a and IL-4 expression in the ischemic hemisphere extracts. Values are the mean ± SD. *P < 0.05 Saline group vs. Control group, ^#^*P* < 0.05 HFSCs group vs. Saline group, ^∆^*P* < 0.05 HFSCs/HGF group vs. HFSCs/Vehicle group, *n* = 6

In order to explore the impact of HFSC treatment on the inflammatory reaction in I/R injured brain, immunohistochemical method was performed for microglia activation and western blot for inflammatory cytokines production. Iba-1 is a microglia-specific calcium-binding protein, which is associated with microglia activation [44]. We analyzed the localization of Iba-1 in the penumbra to assess microglia activation in all groups (Fig. 4A–E). The number of Iba-1-positive cells significantly increased at 7 days after cell transplantation, whereas HFSC treatment inhibited microglia activation. Furthermore, as shown in Fig. 4F, the number of Iba-1-positive cells was significantly lower in the HFSCs/HGF group compared to those in the HFSCs group and HFSCs/Vehicle group (*P* < 0.05).

Among the pro-inflammatory cytokines stands out one classical cytokine, TNF-α, which has been demonstrated as an important pro-inflammatory cytokine in several I/R models, including cerebral I/R injury [45, 46]. In mice cerebral I/R model, an increased expression of TNF-α is detected in the ipsilateral hemisphere along with increased BBB permeability which can be significantly inhibited by TNF-α antibody [47, 48]. On the other hand, IL-4 is believed as an anti-inflammatory cytokine in many pathological conditions and plays a protective role against ischemic stroke [49, 50]. Interleukin 4 receptor alpha chain (IL-4Ra) is expressed in neurons and plays a critical role in modulating neuronal death through activation of signal transducer and activator of transcription 6 (STAT6) during ischemia [51]. We extracted whole proteins from ischemic hemisphere tissue and determined these cytokines expression by western blot at 7 days after cell transplantation (Fig. 4G). The expression of TNF-α and IL-4 both increased in the Saline group than those in the Control group, whereas TNF-α expression was down-regulated and IL-4 was elevated by HFSCs treatment. In addition, HFSCs/HGF group showed a stronger effect compared with HFSCs and HFSCs/Vehicle groups (*P* < 0.05) (Fig. 4H and I). These results indicated HFSCs could regulate the inflammatory reaction by influencing microglia activation and cytokines production.

### HFSC transplantation protected BBB integrity

**Fig. 5:**
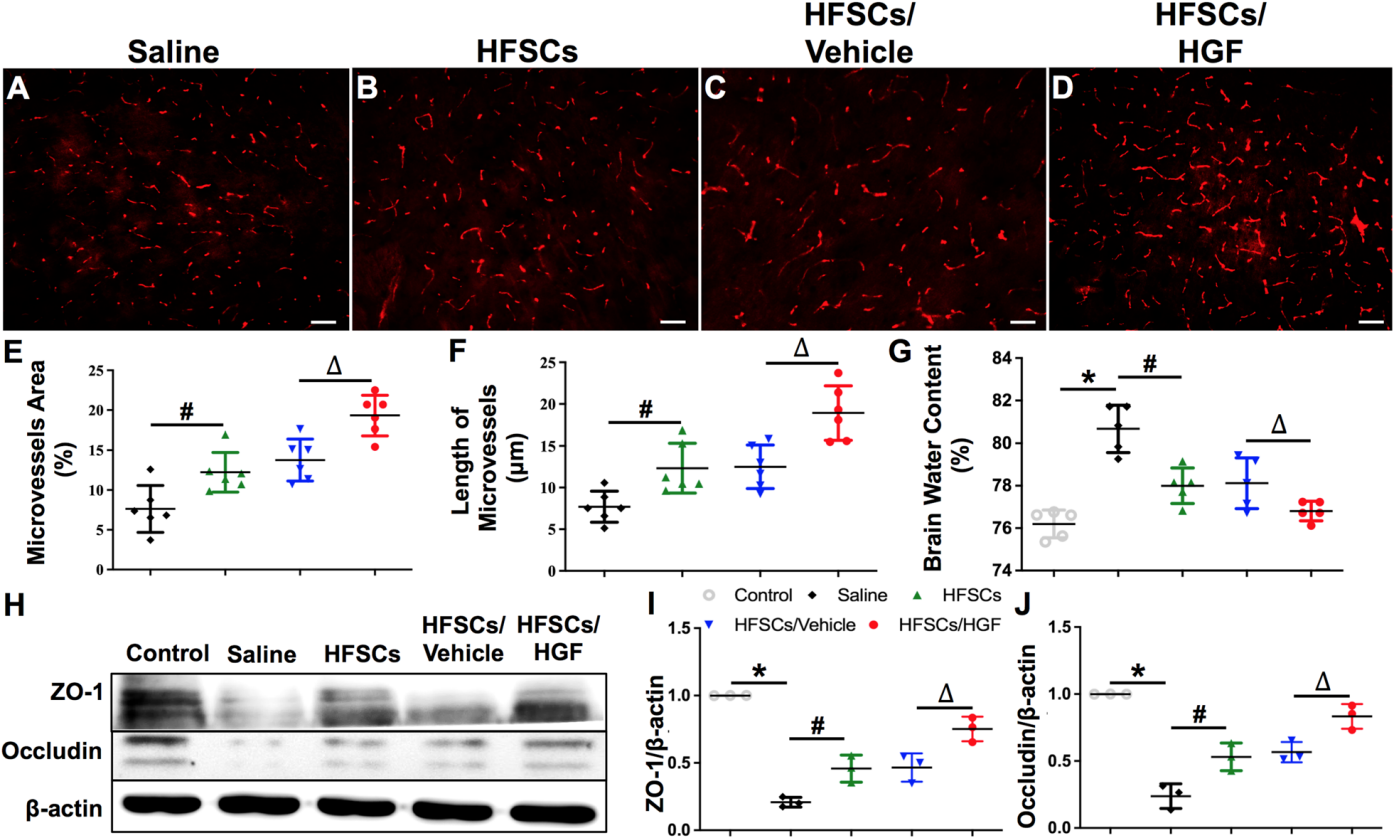
HFSCs and HFSCs/HGF promoted angiogenesis and protected blood–brain barrier. **A–D** Immunofluorescent staining of vWF indicating microvessels in the penumbra. Scale bar = 50 µm. **E** and **F** The quantitative analysis of the density and length of microvessels. **G** The percentage of water content in all groups. **H** Western blot analysis of the tight junction proteins in the ischemic hemispheres. **I** and **J** The quantitative analysis of the ratios of ZO-1 and occludin to β-actin, respectively. Values are the mean ± SD. *P < 0.05 Saline group vs. Control group, ^#^*P* < 0.05 HFSCs group vs. Saline group, ^∆^*P* < 0.05 HFSCs/HGF group vs. HFSCs/Vehicle group, *n* = 6

The densities and morphology of microvessels around the ischemic zone were evaluated using immunofluorescent staining of anti-vWF antibody, an endothelial cell marker (Fig. 5A–D). The microvessels density and length of vascular wall were significantly higher in the HFSCs/HGF group compared to those in the HFSCs and HFSCs/Vehicle groups at 7 days after cells transplantation (Fig. 5E, F) (*P* < 0.05). The results of brain water content in different groups are exhibited in Fig. 5G. The increase of water content after cerebral I/R was attenuated by HFSCs treatment. HFSCs/HGF showed a stronger therapeutic effect and HFSCs/Vehicle exhibited equivalent ability compared to HFSCs group (*P* < 0.05).

BBB insult begins early even before the onset of neuronal damage and influences the extent of brain injury [52, 53]. Tight junction between adjacent endothelial cells is the key element in the BBB disruption during ischemic stroke [54]. The tight junction protein expression in the ischemic hemisphere was evaluated by western blot at 7 days after cell transplantation (Fig. 5H). Compared with the Control group, the expression of occluding and ZO-1 was drastically decreased in the Saline group. While a significant increase in the levels of ZO-1 and occluding expression were seen in HFSCs/HGF group compared to those in HFSCs and HFSCs/Vehicle groups (Fig. 5I, J) (*P* < 0.05). These data show that HFSCs played a role in promoting angiogenesis and protecting BBB integrity as well.

### HGF enhanced HFSCs differentiation potential

**Fig. 6:**
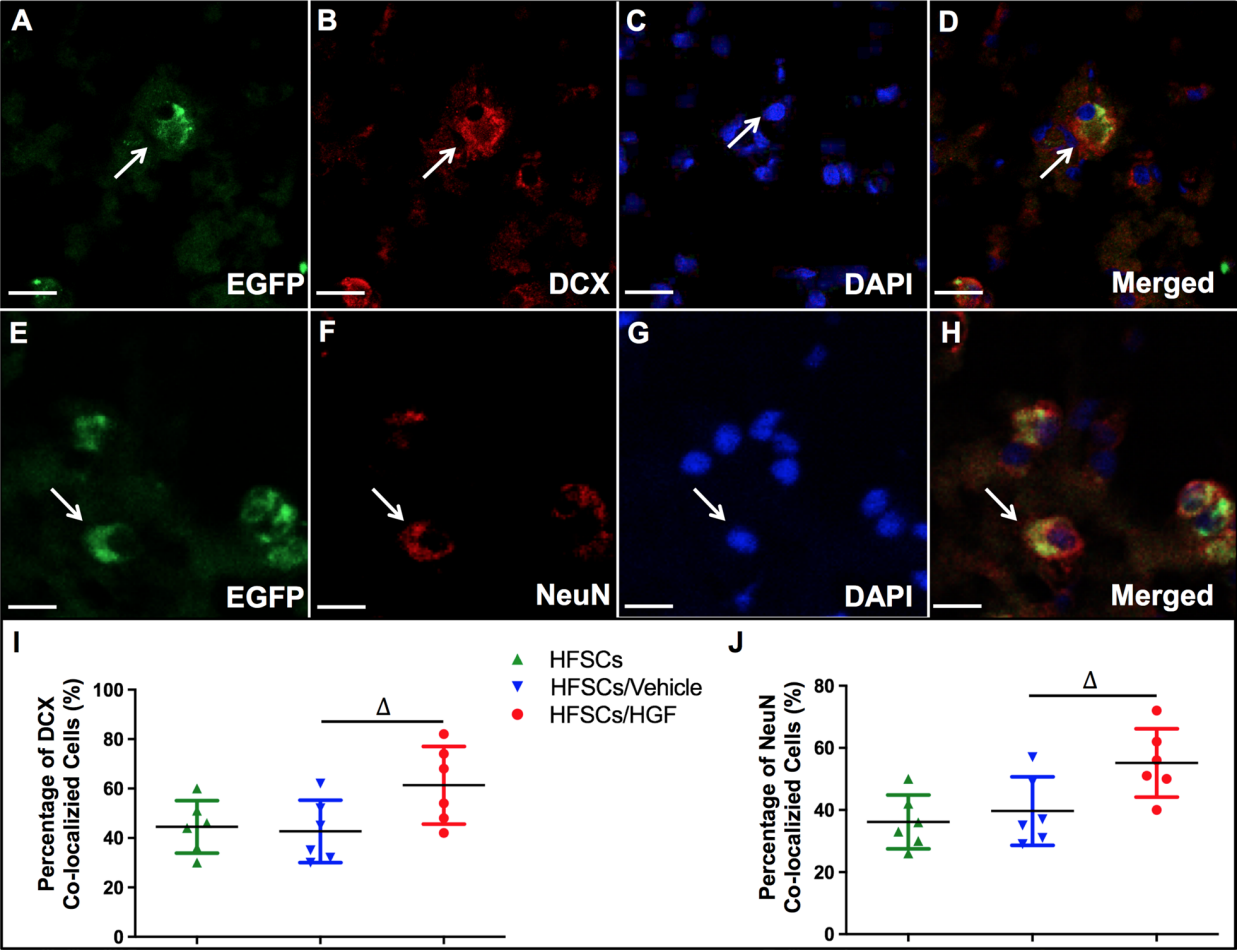
HGF promoted HFSCs differentiation. **A** and **E** The cell showing green fluorescence represents HFSCs that were transduced with the viral vector carrying HGF gene. **B** and **F** Immunofluorescent staining of DCX and NeuN with red fluorescence. **C** and **G** DAPI staining emitted blue fluorescence at 405 nm. **D** and **H** The merged images of EGFP, DCX or NeuN and DAPI. Bar = 10 µm. **I** and **J** The statistical analysis of the percentages of double-stained HFSCs in total EGFP-expressing HFSCs in different groups. Values are the mean ± SD. *P < 0.05 Saline group vs. Control group, ^#^*P* < 0.05 HFSCs group vs. Saline group, ^∆^*P* < 0.05 HFSCs/HGF group vs. HFSCs/Vehicle group, *n* = 6

The spatial distribution of HFSCs was observed and analyzed following transplantation. The grafted EGFP-expressing HFSCs or PKH-67 labeled HFSCs could be identified easily under fluorescent microscope, and they visibly gathered in the ischemic boundary zone at 2 weeks after transplantation, but rarely migrated to normal hemisphere (not shown) [55]. To further investigate the presence of HFSCs, immunofluorescence labelling was performed for neural specific markers. Excitedly, EGFP or PHK-67 labelled cells (green fluorescent) were found to express neuron-specific markers DCX (Fig. 6A–D) and NeuN (Fig. 6E–H), indicating the potential development of the HFSCs into neuron-like cells. We analyzed and compared the number of co-localization cells with neuron-specific markers in three groups (Additional file 3: Fig. S3). HFSCs/HGF group showed an increased number of co-localization cells than HFSC group and HFSCs/Vehicle group (Fig. 6I, J) (*P* < 0.05).

Therefore, the evidence supports that intravenous HFSC transplantation in the acute phase of I/R improved neurological outcome and decreased infarct volume. Moreover, the combination with HGF enhanced their therapeutic effects.

## Discussion

In this study, we demonstrated for the first time HGF modified HFSCs could significantly improve the neurological outcome of cerebral I/R injury by inflammation alleviation, BBB protection, angiogenesis promotion and neuron apoptosis inhibition. The inflammatory response caused by cerebral I/R is characterized by rapid activation of microglia, accumulation of inflammatory cytokines, and infiltration of inflammatory cells into damaged brain tissues [56]. Microglia are the resident immunocompetent cells of the CNS with phagocytic ability which will be activated and recruited to the damaged area and release pro-inflammatory cytokines such as TNF-α, IL-1, and IL-6 in the acute phase [57, 58]. IL-4, as a constitutively expressed anti-inflammatory cytokine, would counterbalance the actions of IL-1β and TNF-α and suppress cytokine receptor expression and activation [59]. Systemic administration of IL-4 could reduce ischemic lesion and improve neurological function after stroke [60–62]. Our results showed HFSC transplantation inhibited the microglia activation and regulated the inflammatory cytokines TNF-α and IL-4 expression (Fig. 4G). Our finding is consistent with studies showing that mesenchymal stem cells (MSCs) can regulate astrocytes activation through paracrine cytokines such as IL-4 and TGF-β [63, 64].

The pro-inflammatory cytokines are also the key mediators of BBB damage in ischemic stroke, since intracerebral administration of TNF-α will cause BBB breakdown [65, 66]. In addition, another pivotal factor in maintaining BBB integrity is tight junction proteins including claudins, occludin, junction adhesion molecules (JAMs), and several cytoplasm accessory proteins such as ZO-1 [67, 68]. After tight junction protein degradation, the macromolecular proteins in circulation, such as albumin, leak throughout impaired BBB and cause edema around capillaries. The mechanical occlusion of capillaries leads to neuron ischemia and hypoxia, finally resulting in neuron apoptosis [69]. Research suggests that MSC transplantation could protect endothelial cells and BBB integration against stroke attack [70, 71], but there is no evidence to suggest HFSCs have a protective effect on BBB disruption. Due to our data, the effect of HFSCs on microglia activation and inflammatory cytokines proves it plays a protective role in BBB disruption. Moreover, HFSCs can also protect BBB integrity by inhibiting the degradation of tight junction protein ocluddin and ZO-1, thus alleviating brain edema (Fig. 5G, H). In addition, angiogenesis in ischemic penumbra plays an important role in the blood and oxygen resupply of brain tissue and the recovery of neurological function [72, 73]. Studies have suggested that MSCs have the ability to enhance angiogenesis by secreting trophic factors, such as VEGF-A, VEGF-C, bFGF, PGF and HGF [74–76]. In present research, we revealed that HFSCs treatment increased the density of microvessels in the penumbra (Fig. 5A–F).

The pathophysiological process of cerebral ischemia is a complex network. Traditional drugs targeting a single molecule are difficult to dynamically regulate the pathophysiological network and obtain ideal curative effect. Stem cell transplantation is considered as a promising biological therapy because of its multiple regulation of pathophysiological pathways and feedback regulation of microenvironments [77–80]. At present, stem cells that can be used to treat CNS diseases mainly include neural stem cells (NSCs), MSCs and induced pluripotent stem cells (iPSCs) [81–83]. However, the clinical application of these stem cells is limited by some difficulties, such as the lack of appropriate donor sources, immune incompatibility and ethical problems [84]. HFSCs have been found derived from the same ectoderm as the nervous system and can differentiate into neurons, glial cells, smooth muscle cells, keratinocytes and melanocytes in vitro [85]. Therefore, they may have promising neural differentiation and neurotrophic ability [20, 86]. Recently studies demonstrated HFSCs could secrete neurotrophic factors NGF, GDNF and BDNF, and their secretion ability is stronger than that of neuron-like cells differentiated by bone mesenchymal stem cells cultured under the same conditions [20, 86]. Moreover, as a conventional skin appendage, each adult has about 5 million hair follicles, which provide an abundant and easily accessible cell source for HFSCs, supporting the exploration of regenerative medicine and stem cell therapy [36].

Hepatocyte growth factor (HGF) is a powerful pleiotropic cytokine, which plays an important role in many biological processes, such as cell proliferation, angiogenesis and anti-apoptosis [22–24]. Recent studies have showed that HGF could protect BBB integrity, attenuate brain edema, promote endogenous repair and functional recovery in rodent models after cerebral ischemia [87]. HGF and its specific receptor c-Met are expressed and functional in vascular endothelial cells and neurons [88]. Shang observed that HGF significantly amplified the angiogenesis following cerebral ischemic stroke [87]. The molecular mechanisms of the angiogenic activity of HGF may be strongly associated with the E-twenty-six (ETS) pathway [89, 90]. Another study shows that HGF/c-Met could phosphorylate STAT3 and then promote Bcl-2 transcription, underlying the anti-apoptotic effect as an upstream signal [91]. In present study, HFSCs overexpressed-HFG played a key role in promoting the neuroprotective and neurological functions recovery of ischemic stroke through BBB protection, angiogenesis promotion and neuron apoptosis inhibition (Figs. 2 and 5). Moreover, the fluorescence confocal data showed that compared with HFSCs group, more fluorescent cells in HGF-overexpressed HFSCs group migrated to the ischemic penumbra 2 weeks after transplantation and expressed neuron specific markers (Additional file 3: Fig. S3). These results suggest that HGF may promote HFSCs homing and neural differentiation, which is consistent with the role of HGF in promoting proliferation, differentiation, and migration of NSCs and MSCs [92–95]. In addition, the homing effect of HFSC may also assists HGF in entering the lesion area of CNS to plays its protective role. These clues suggest that HGF and HFSCs may play a synergistic work rather than a simple superposition of their curative effects.

## Conclusions

**Fig. 7:**
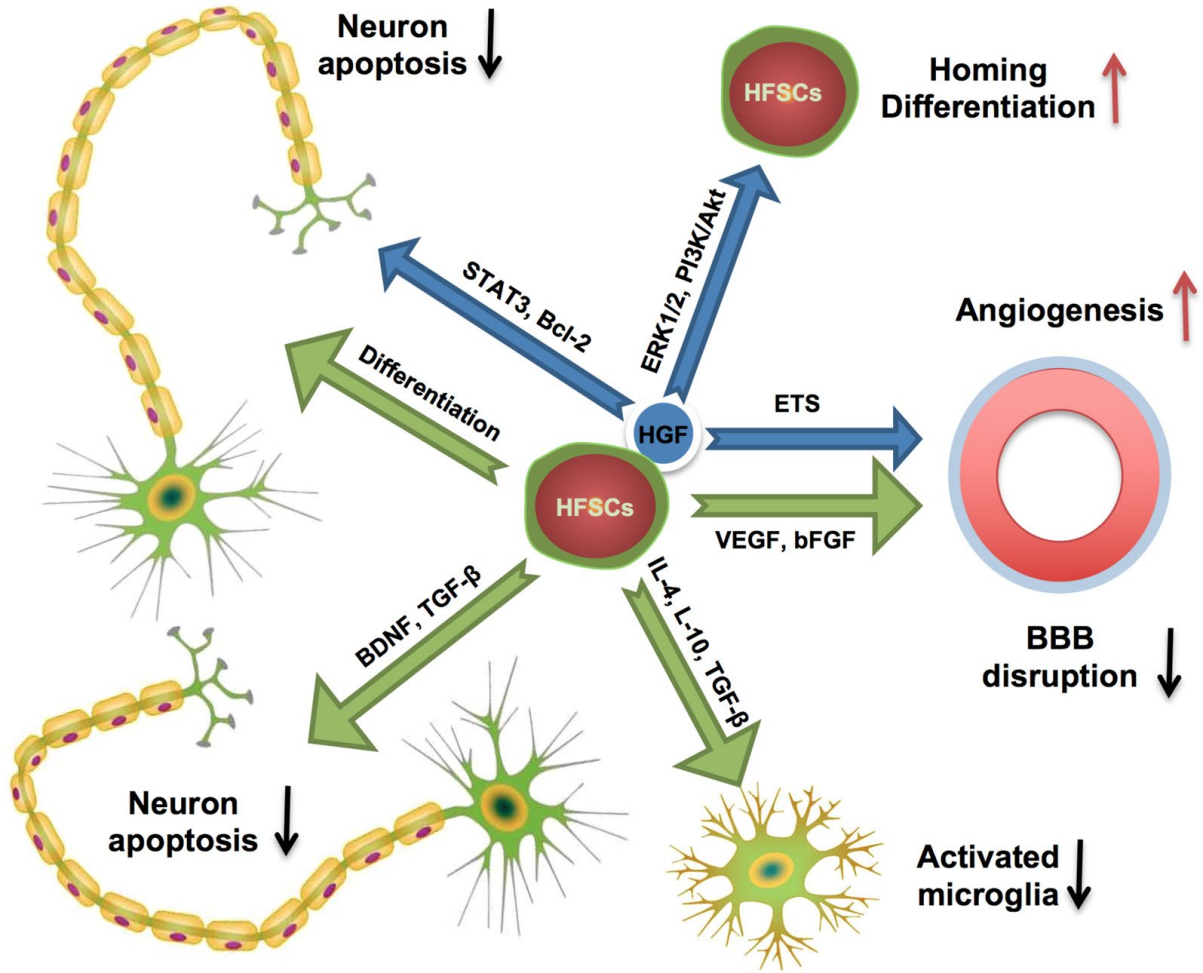
Summary of the therapeutic effects of HFSCs and HFSCs/HGF treatment on I/R induced brain damage

In summary, our results firstly demonstrated HFSCs, as an abundant and available stem cell type, played a role in alleviating inflammation, protecting BBB integration, promoting angiogenesis and eventually improved neurological recovery in acute phase after cerebral I/R injury. The combination of HFSCs and HGF has synergistic effect which enhanced the therapeutic benefit (Fig. 7). Stem cells have been considered as a prospective therapy in stroke due to the neuroprotective and modulate inflammatory effects on neurological disorders [77–80]. Although further study is needed to elucidate the dynamic changes and the long-term effects of HFSCs transplantation for the treatment of stroke, the present study shed a light on the widely application of stem cell therapy in stroke.

## Supplementary Information

**Additional file 1: Figure S1** Morphology and antigenic phenotyping of the HFSCs modified with the rat hepatic growth factor gene (HGF). (**A**) The original cell formation of HFSCs. (**B–C**) The first and third progeny of cultured HFSCs exhibited plastic adherence, colony formation, paving stone-like morphology. (**D**) Construction of the lentiviral vector that contains the rat HGF gene and green fluorescent protein reporter gene (EGFP). (**E**) The dynamic changes between transduction efficiency and multiplicity of infection (MOI). (**F**) Western blot analysis of HGF expression in the culture medium. (**G**) The transduced HFSCs express bright green fluorescence. Bar = 100 µm. (**H, I**) Osteogenic and adipogenic differentiations. Bar = 100 µm. (**J–M**) Mesenchymal stem cells surface markers expression of fluorescence-activated cell sorting (FACS) analysis.

**Additional file 2: Figure S2** MCAO model establishment and assessment. (**A**) Serial coronal slices of healthy rat brain. (**B**) Consecutive coronal slices of I/R rat brain. Typical photographs of rat brain stained with 2,3,5-Triphenyltetrazolium chloride (TTC), wherein no infarction tissue was stained red, while the infarct tissue unstained (white color). Scale bar = 20 mm. (**C, D**) Nissl staining of MCAO models revealed lesions in the brain tissues with diminished numbers of neurons and chaotic neuronal configuration. Scale bar = 5 mm. **(E, F**) Enlargement of healthy area and infarcted area. The double-arrow indicates nissl-positive neurons. The arrow indicates nuclear pyknosis with karyorrhexis. Scale bar = 100 µm. Values are the mean ± SD. ^∆^*P* < 0.05 vs. HFSCs group, *n* = 6.

**Additional file 3: Figure S3** The representative images of co-localization of PKH67 or EGFP and neuron-specific markers. (**A, E, I, M, Q, U**) The first column shows HFSCs. (**B, F, J, N, R, V**) The second column shows DCX or NeuN-positive cells. (**C, G, K, O, S, W**) The third column shows nucleus stained by DAPI. (**D, H, L, P, T, X**) The fourth column shows the merged pictures of first three columns. Scale bar = 50 µm.

**Additional file 4: Table S1** Neurological scoring system.

**Additional file 5.** Supplementary results.

## Data Availability

The datasets generated during the current study are available from the corresponding authors upon reasonable requests.

## Abbreviations

I/R: Ischemia/reperfusion
HFSCs: Hair follicle stem cells
HGF: Hepatocyte growth factor
BBB: Blood–brain barrier
CNS: Central nervous system
MCAO: Middle cerebral artery occlusion
TTC: 2,3,5-Triphenyltetrazolium chloride
NeuN: Neuron-specific nuclear protein
Iba-1: Ionized calcium-binding adaptor molecule 1
DAPI: 4’-6-Diamidino-2-phenylindole
ZO-1: Zonula occluden 1
MSCs: Mesenchymal stem cells
