# Supplementary figures and images for: Hepatocyte growth factor-modified hair follicle stem cells ameliorate cerebral ischemia/reperfusion injury in rats
Source: Stem Cell Res Ther. 2023 Feb 13;14:25. doi: 10.1186/s13287-023-03251-5 (PMC9926795; doi:10.1186/s13287-023-03251-5)

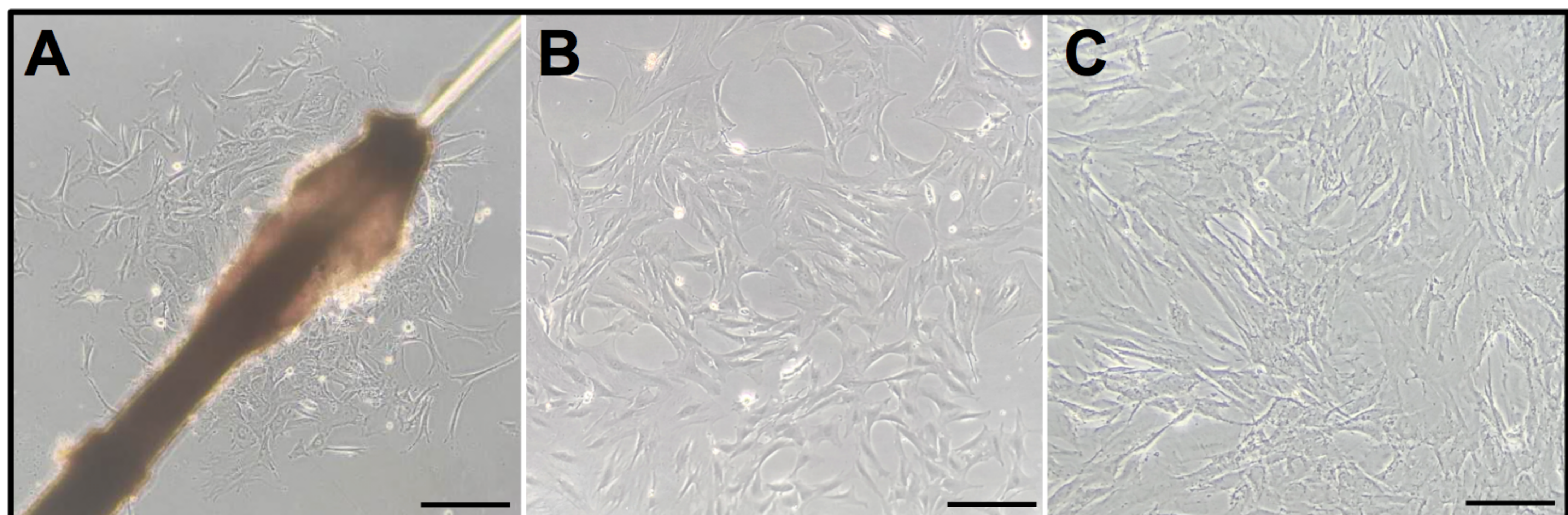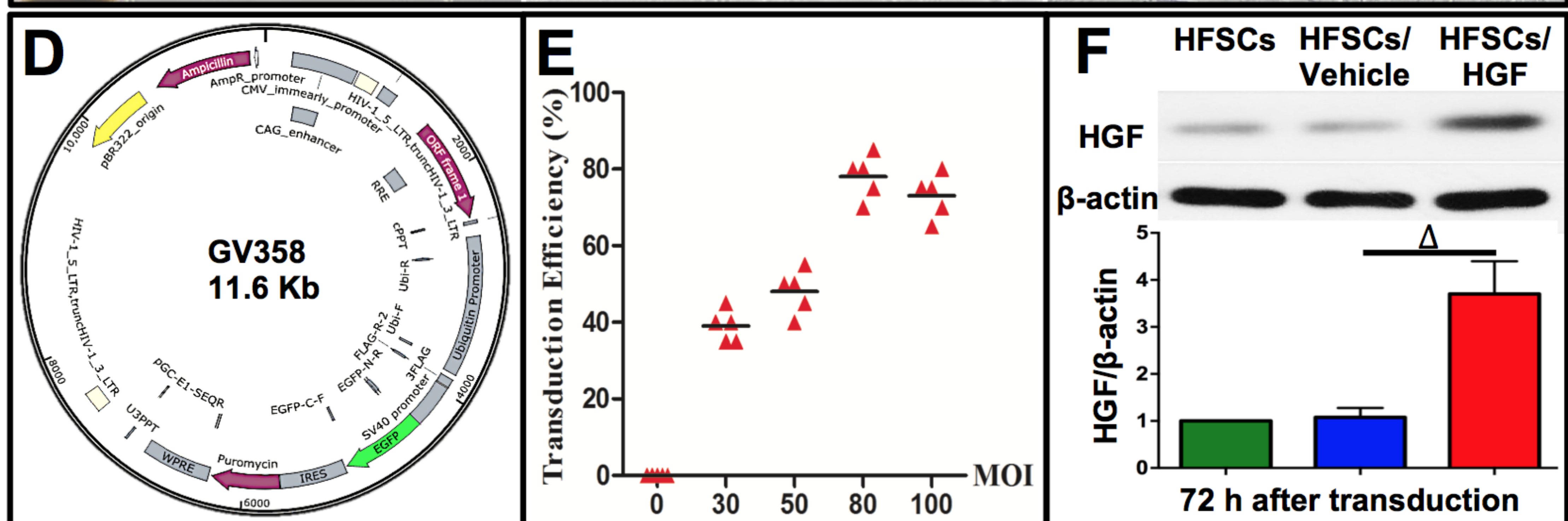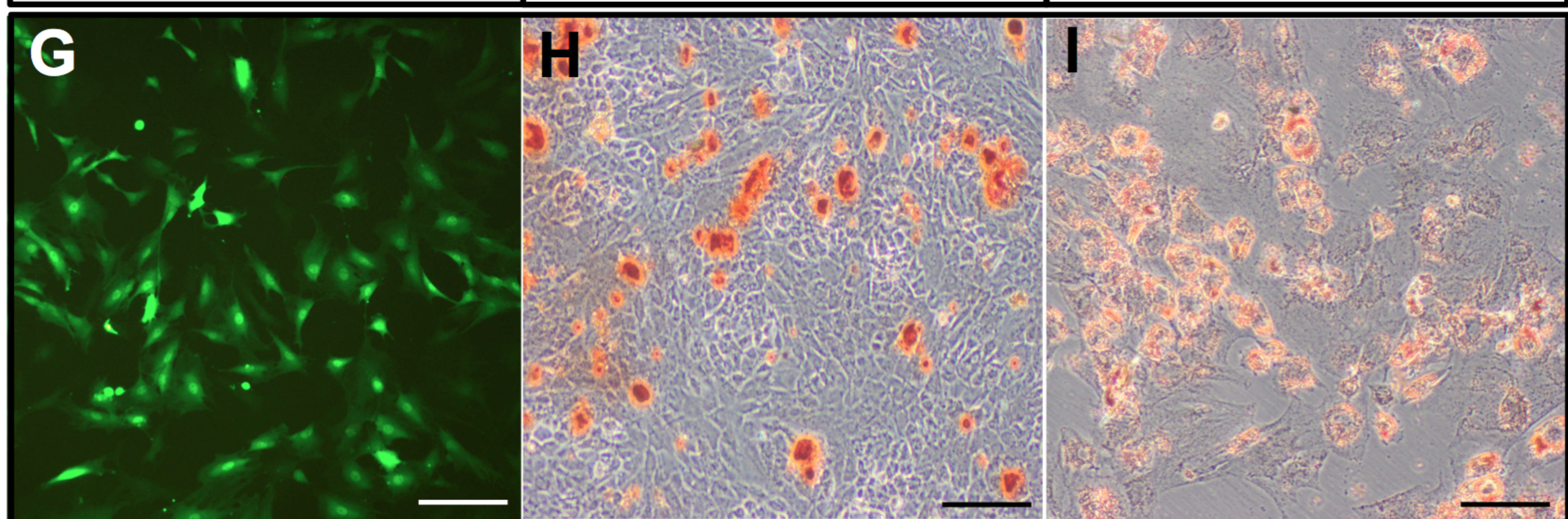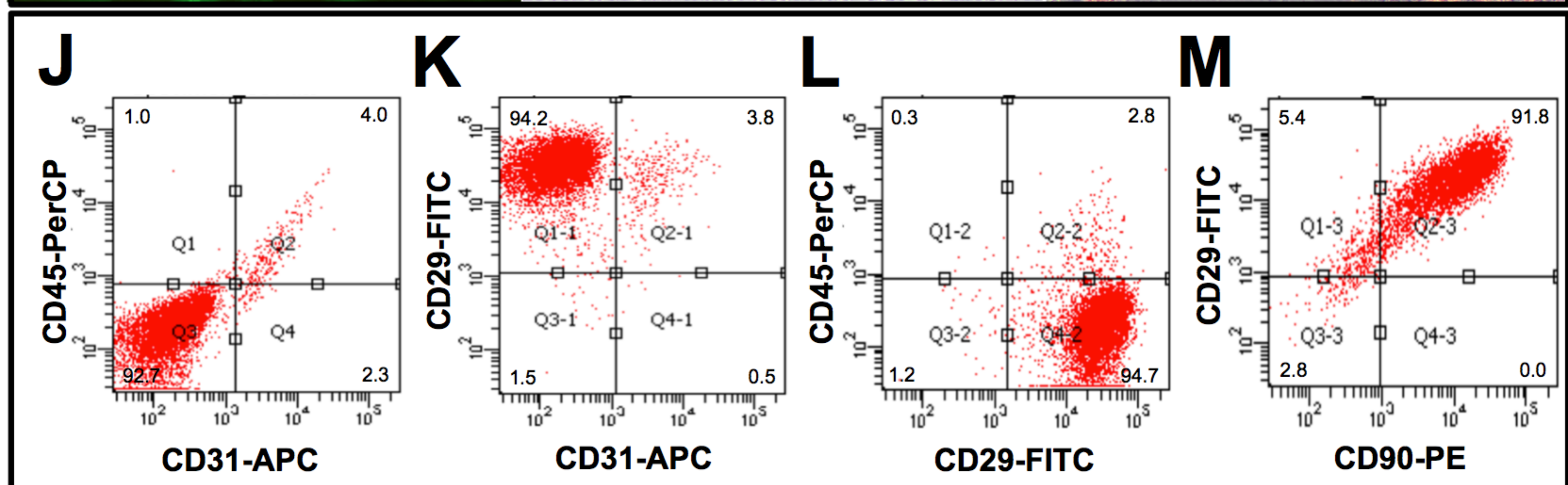

Supplement: Supplementary file 1 — Additional file 1: Figure S1 Morphology and antigenic phenotyping of the HFSCs modified with the rat hepatic growth factor gene (HGF). (A) The original cell formation of HFSCs. (B–C) The first and third progeny of cultured HFSCs exhibited plastic adherence, colony formation, paving stone-like morphology. (D) Construction of the lentiviral vector that contains the rat HGF gene and green fluorescent protein reporter gene (EGFP). (E) The dynamic changes between transduction efficiency and multiplicity of infection (MOI). (F) Western blot analysis of HGF expression in the culture medium. (G) The transduced HFSCs express bright green fluorescence. Bar = 100 µm. (H, I) Osteogenic and adipogenic differentiations. Bar = 100 µm. (J–M) Mesenchymal stem cells surface markers expression of fluorescence-activated cell sorting (FACS) analysis. [file 13287_2023_3251_MOESM1_ESM.pdf]

**A**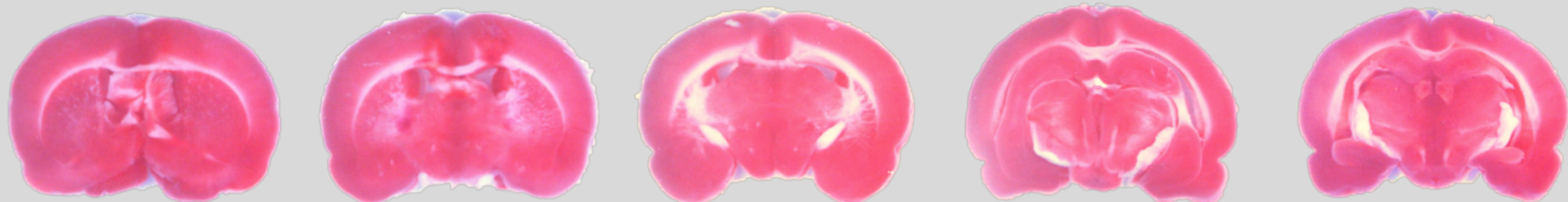**B**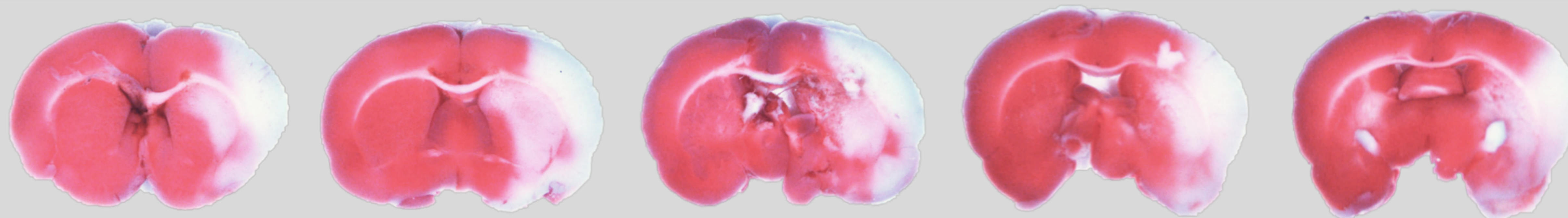**C**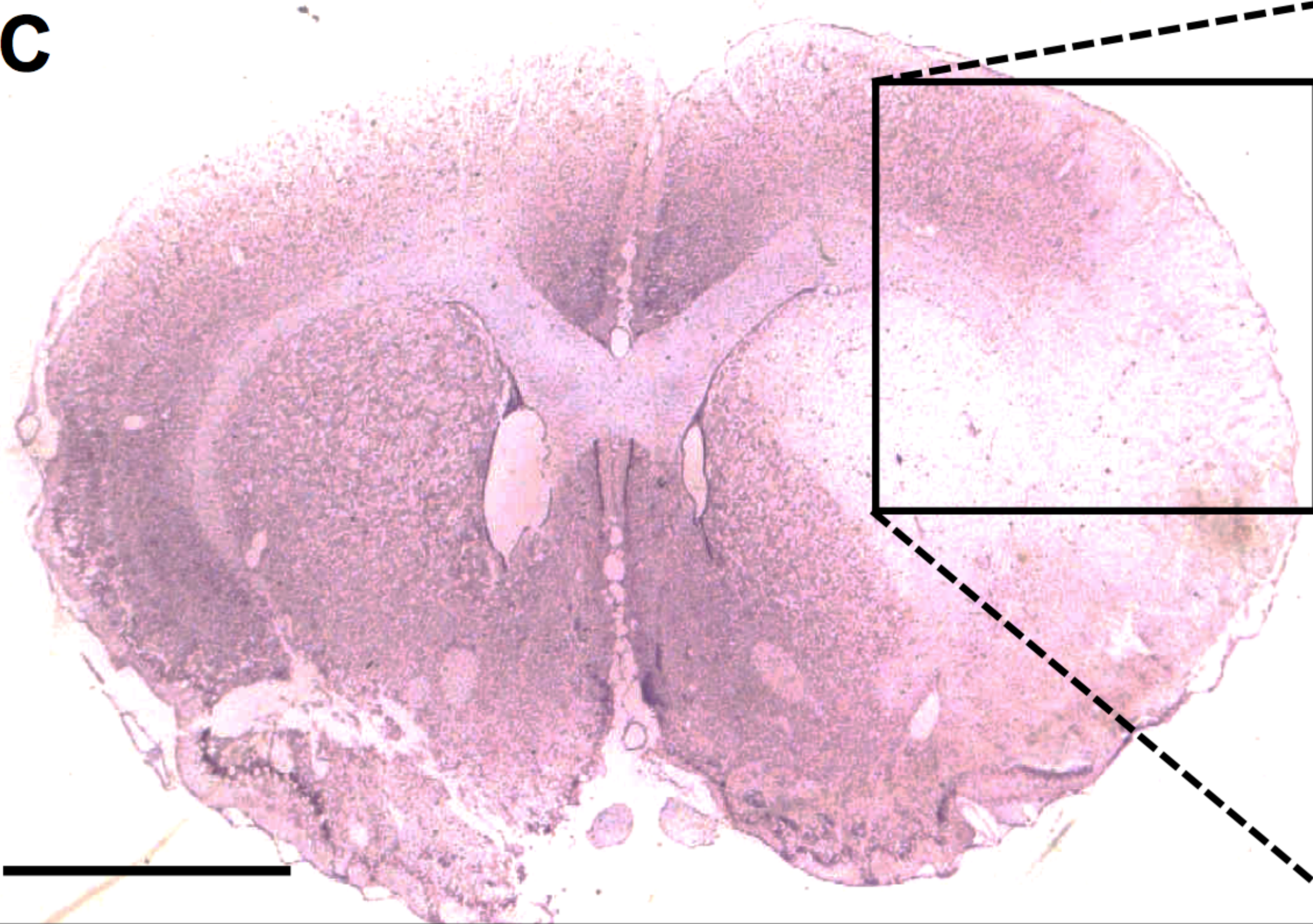**D**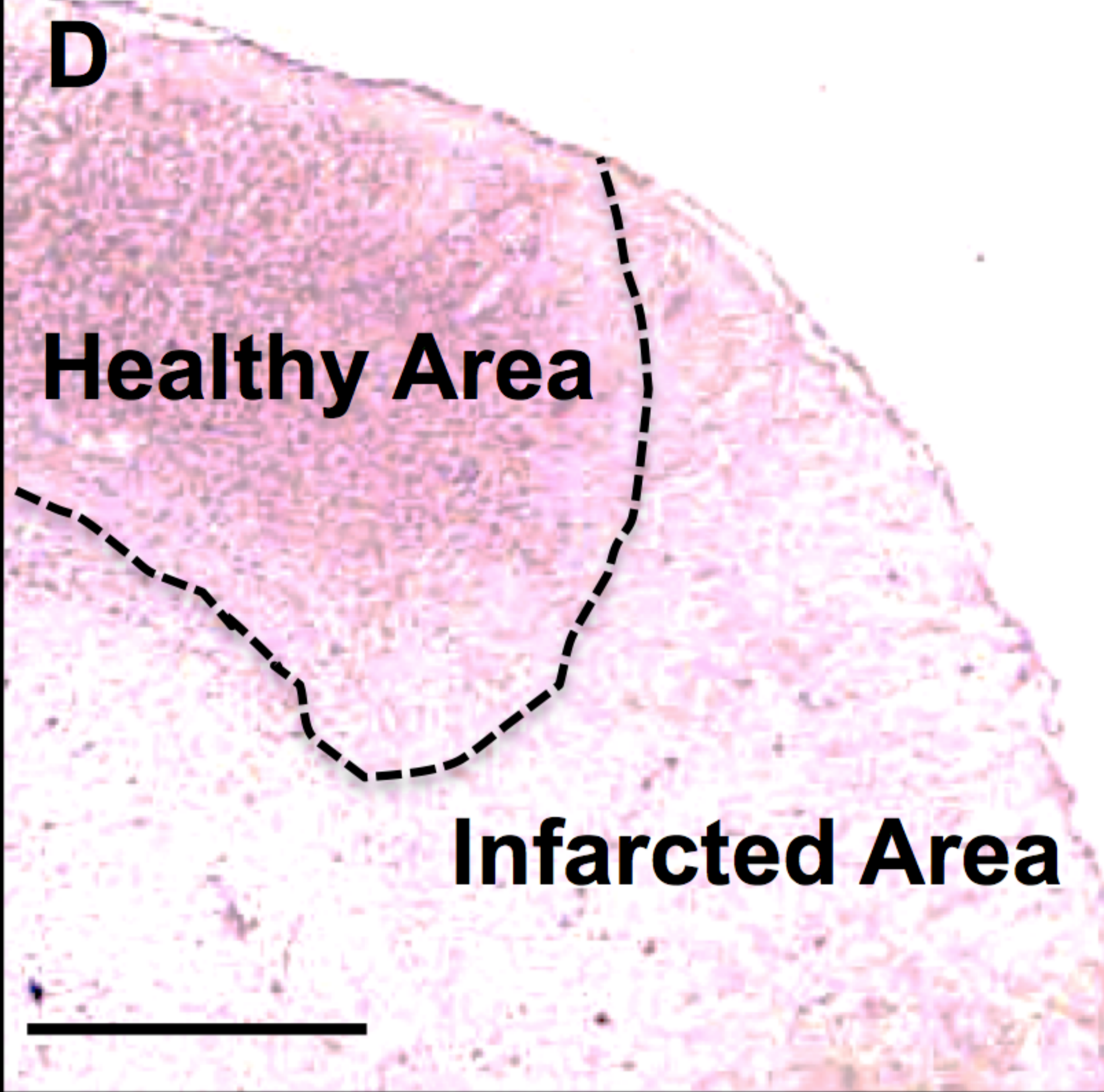**E**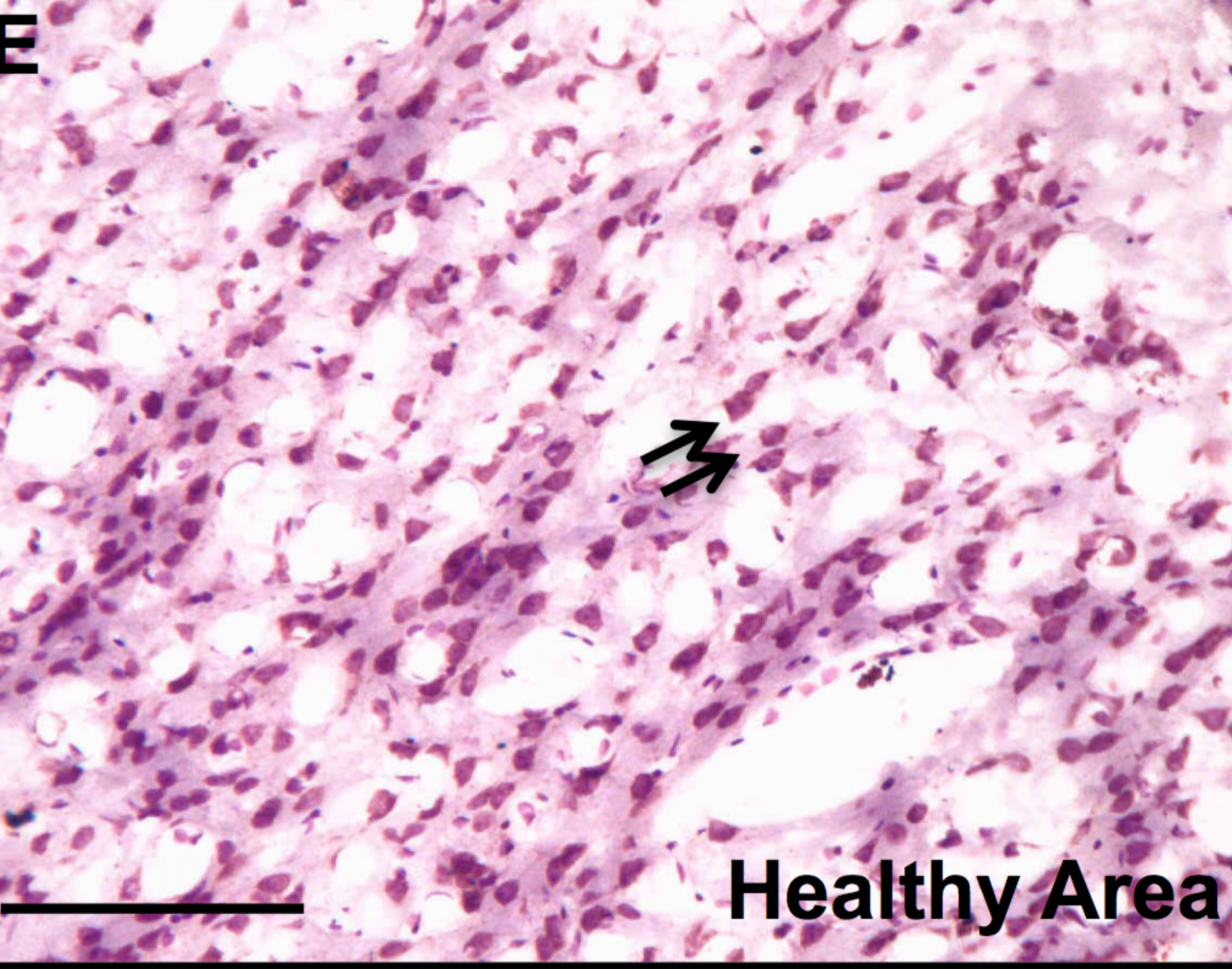**F**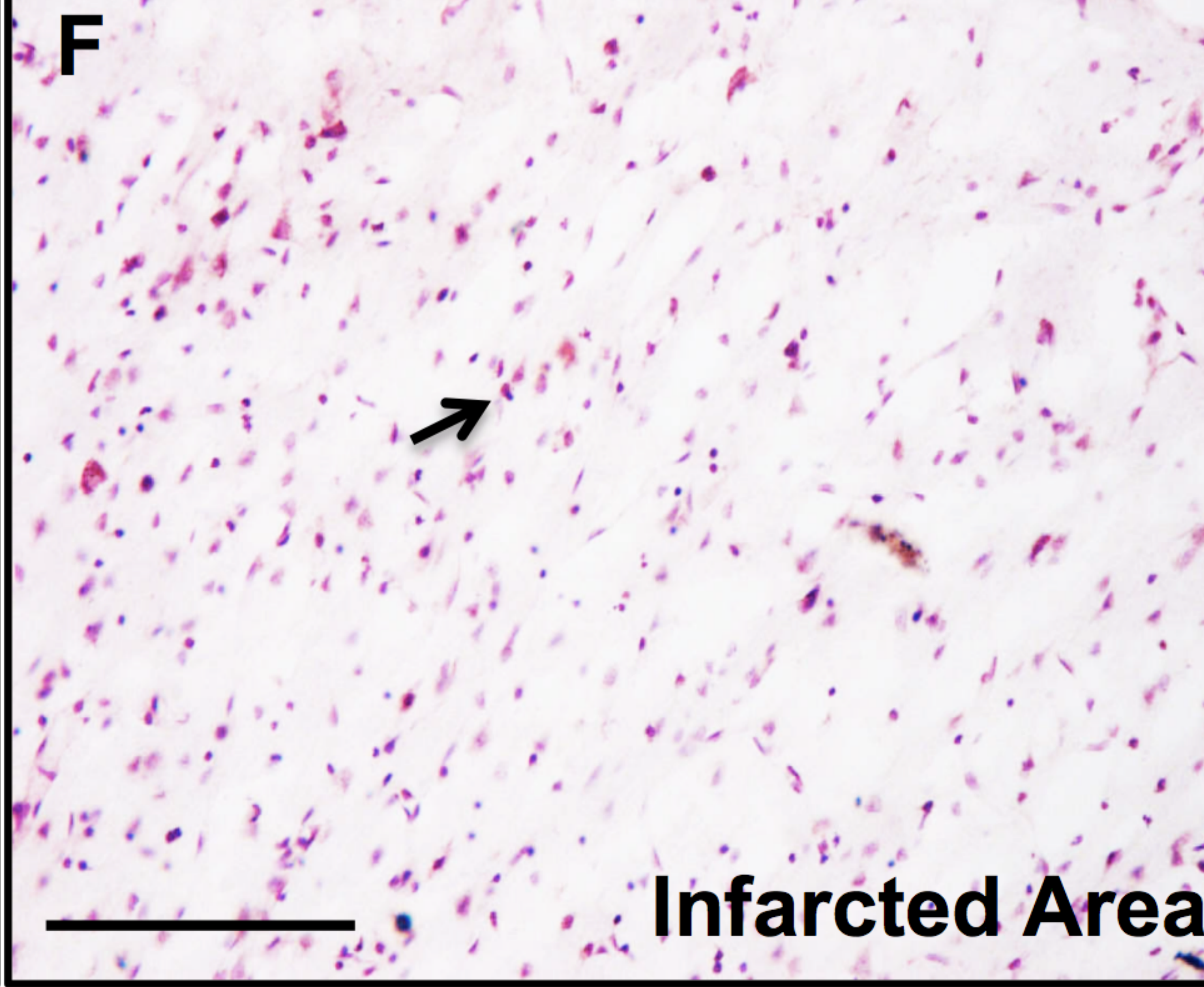

Supplement: Supplementary file 2 — Additional file 2: Figure S2 MCAO model establishment and assessment. (A) Serial coronal slices of healthy rat brain. (B) Consecutive coronal slices of I/R rat brain. Typical photographs of rat brain stained with 2,3,5-Triphenyltetrazolium chloride (TTC), wherein no infarction tissue was stained red, while the infarct tissue unstained (white color). Scale bar = 20 mm. (C, D) Nissl staining of MCAO models revealed lesions in the brain tissues with diminished numbers of neurons and chaotic neuronal configuration. Scale bar = 5 mm. (E, F) Enlargement of healthy area and infarcted area. The double-arrow indicates nissl-positive neurons. The arrow indicates nuclear pyknosis with karyorrhexis. Scale bar = 100 µm. Values are the mean ± SD. ∆P < 0.05 vs. HFSCs group, n = 6. [file 13287_2023_3251_MOESM2_ESM.pdf]

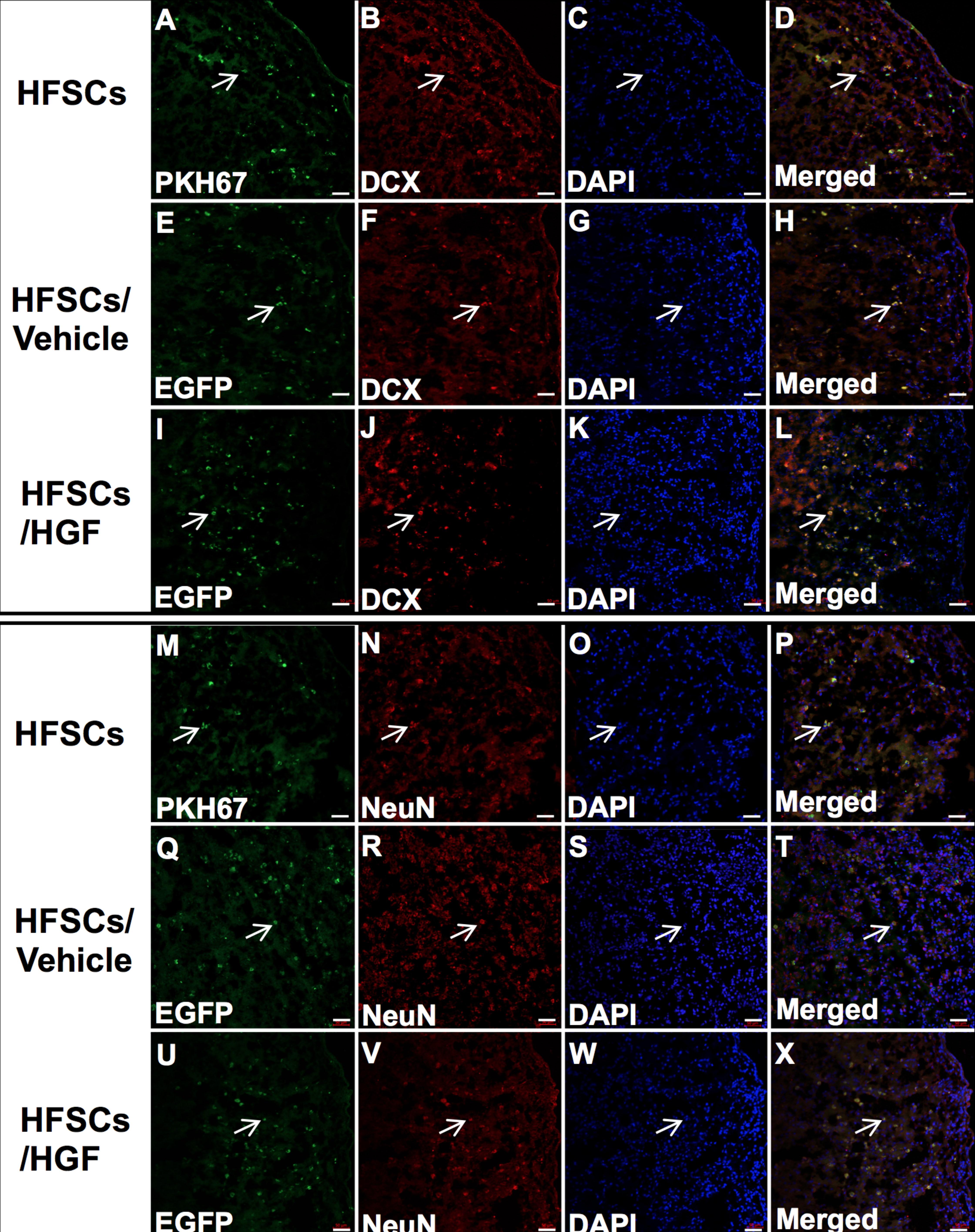

Supplement: Supplementary file 3 — Additional file 3: Figure S3 The representative images of co-localization of PKH67 or EGFP and neuron-specific markers. (A, E, I, M, Q, U) The first column shows HFSCs. (B, F, J, N, R, V) The second column shows DCX or NeuN-positive cells. (C, G, K, O, S, W) The third column shows nucleus stained by DAPI. (D, H, L, P, T, X) The fourth column shows the merged pictures of first three columns. Scale bar = 50 µm. [file 13287_2023_3251_MOESM3_ESM.pdf]
