# Supplementary material for: Hepatocyte growth factor-modified hair follicle stem cells ameliorate cerebral ischemia/reperfusion injury in rats
Source: Stem Cell Res Ther. 2023 Feb 13;14:25. doi: 10.1186/s13287-023-03251-5 (PMC9926795; doi:10.1186/s13287-023-03251-5)
Supplement: Supplementary file 4 — Additional file 4: Table S1 Neurological scoring system. [file 13287_2023_3251_MOESM4_ESM.pdf]

Table 1 Neurological scoring system

	Score			
	0	1	2	3
Spontaneous activity (3 min test period)	No movement	Slight movement	Touches 1 or 2 sides of cage	Touches 3 or 4 sides of cage
Symmetry of movement (forelimbs and hind limbs)	Total asymmetry	Near-total asymmetry	Mild asymmetry	Complete symmetry
Floor walking	No walking	Walks in circles only	Curvilinear path	Straight path
Beam walking	Falls off of beam	Hugs beam	Stands on beam	Walks on beam
Response to vibrissae touch of left side		No response	Weak response	Symmetrical response
