# Supplementary material for: Hepatocyte growth factor-modified hair follicle stem cells ameliorate cerebral ischemia/reperfusion injury in rats
Source: Stem Cell Res Ther. 2023 Feb 13;14:25. doi: 10.1186/s13287-023-03251-5 (PMC9926795; doi:10.1186/s13287-023-03251-5)
Supplement: Supplementary file 5 — Additional file 5. Supplementary results. [file 13287_2023_3251_MOESM5_ESM.docx]

**Supplementary results**

**Virus-incorporated HFSCs exhibited mesenchymal stem cell antigenic markers**

Cultured HFSCs exhibited plastic adherence, colony formation, paving stone-like morphology (*Figure S1 A-C*). The construction of the lentivirus vector that contains rat HGF gene and green fluorescent protein reporter gene (EGFP) is shown in *Figure S1 D*. The EGFP gene construct in the viral vector were used to assess the transduction efficiency of the HGF-expressing virus in cultured HFSCs. The majority of transduced HFSCs emitted bright green fluorescence (*Figure S1 G*). The transduction efficiency was evaluated by the ratio of EGFP-positive cells to the total cell number. The transduction efficiency was 80% at the MOI of 80 (*Figure S1 E*). The expression of HGF was 3.7 times in the medium of HFSCs/HGF than HFSCs, which were determined by western blot after cultured at 1 x 10^6^ cells per mL for 72 h (n=3) (*Figure S1* *F*).

Osteogenic and adipogenic differentiation induction was performed to assess the multipotency of the cultured HFSCs. After 2 weeks of culture in osteogenic and adipogenic differentiation media, the cells differentiated into osteogenic and adipogenic lineages, as demonstrated through Alizarin red (*Figure S1 H*) and Oil Red O staining (*Figure S1 I*). To detect the expression of various surface markers on the isolated cells, FACS analysis was carried out. The FACS results indicated that the cells mainly expressed CD29 and CD90, which are surface markers of mesenchymal stem cells, but barely expressed CD31and CD45, the surface antigens of endothelial cells and leucocytes, respectively (*Figure S1 J-M*).

**Focal cerebral ischemia/reperfusion animal model establishment**

TTC and Nissl staining was used to confirm and analyze the focal cerebral I/R model. Normal brain tissue was stained red by TTC (*Figure S2 A*). Whereas infarcted tissue caused by I/R injury was indicated by white area (*Figure S2 B*). Nissl staining showed remarkable damaged area in accordance with TTC staining (*Figure S2 C and D*). Neurons in the healthy area exhibited normal morphological features (*Figure S2 E*), whereas diverse neuronal damages occurred in the infarcted area, such as cell loss, cell swelling, nuclear pyknosis, and karyorrhexis (*Figure S2 F*). The images of histological staining in this article were taken around the healthy/infarcted boundary.

**Supplementary figure legends**

**Supplementary figure 1.** Morphology and antigenic phenotyping of the HFSCs modified with the rat hepatic growth factor gene (HGF). (**A**) The original cell formation of HFSCs. (**B-C**) The first and third progeny of cultured HFSCs exhibited plastic adherence, colony formation, paving stone-like morphology. (**D**) Construction of the lentiviral vector that contains the rat HGF gene and green fluorescent protein reporter gene (EGFP). (**E**) The dynamic changes between transduction efficiency and multiplicity of infection (MOI). (**F**) Western blot analysis of HGF expression in the culture medium. (**G**) The transduced HFSCs express bright green fluorescence. Bar = 100 μm. (**H-I**) Osteogenic and adipogenic differentiations. Bar = 100 μm. (**J-M**) Mesenchymal stem cells surface markers expression of fluorescence-activated cell sorting (FACS) analysis.

**Supplementary figure 2.** MCAO model establishment and assessment. (**A**) Serial coronal slices of healthy rat brain. (**B**) Consecutive coronal slices of I/R rat brain. Typical photographs of rat brain stained with 2,3,5-Triphenyltetrazolium chloride (TTC), wherein no infarction tissue was stained red, while the infarct tissue unstained (white color). Scale bar = 20 mm. (**C-D**) Nissl staining of MCAO models revealed lesions in the brain tissues with diminished numbers of neurons and chaotic neuronal configuration. Scale bar = 5 mm. **(E-F**) Enlargement of healthy area and infarcted area. The double-arrow indicates nissl-positive neurons. The arrow indicates nuclear pyknosis with karyorrhexis. Scale bar = 100 μm. Values are the mean ± SD. ^∆^*P* < 0.05 vs. HFSCs group, n = 6.

**Supplementary figure 3.** The representative images of co-localization of PKH67 or EGFP and neuron-specific markers. (**A, E, I, M, Q, U**) The first column shows HFSCs. (**B, F, J, N, R, V**) The second column shows DCX or NeuN-positive cells. (**C, G, K, O, S, W**) The third column shows nucleus stained by DAPI. (**D, H, L, P, T, X**) The fourth column shows the merged pictures of first three columns. Scale bar = 50 μm.
